# Patient Activation, Patient‐Physician Communication Quality, and Cancer‐Related Fatigue: A Longitudinal Study of Cancer Survivors

**DOI:** 10.1002/pon.70542

**Published:** 2026-07-14

**Authors:** Duc Truong Nguyen, Martina E. Schmidt, Marlena Milzer, Anna S. Wagner, Senta Kiermeier, Imad Maatouk, Karen Steindorf

**Affiliations:** ^1^ Division of Physical Activity, Cancer Prevention and Survivorship German Cancer Research Center (DKFZ) and National Center for Tumor Diseases (NCT) Heidelberg a Partnership Between DKFZ and University Medical Center Heidelberg Heidelberg Germany; ^2^ Department of Internal Medicine II Chair of Integrated Psychosomatic Medicine and Psychotherapy University Hospital Würzburg Würzburg Germany; ^3^ Department of Psychosomatic Medicine and Psychotherapy Central Institute of Mental Health Medical Faculty Mannheim/Heidelberg University Mannheim Germany; ^4^ University Medical Center Mannheim Mannheim Cancer Center Mannheim Germany

**Keywords:** cancer‐related fatigue, cancer survivors, communication, oncology, patient participation, patient‐physician relationship, self‐management, survivorship

## Abstract

**Objective:**

Cancer‐related fatigue (CRF) is highly prevalent and often insufficiently addressed in routine survivorship care. This study examined whether patient activation and patient‐physician communication quality in early survivorship are associated with subsequent CRF among cancer survivors.

**Methods:**

The observational German LIFT study enrolled 1175 adult survivors of various cancer types. CRF was assessed 4–6, 9, 12, and 24 months post‐diagnosis using the EORTC QLQ‐FA12. Patient activation was assessed with the German Patient Activation Measure (PAM‐13‐D (PAM)) and communication quality with an exploratory five‐item scale (ComQ) 9 months after diagnosis. Multiple linear regression models examined associations of PAM and ComQ with physical and total CRF 12 months (*n* = 1132) and 24 months (*n* = 1067) after diagnosis, adjusting for sociodemographic and clinical covariates. Sensitivity analyses additionally adjusted for previous CRF 4–6 months after diagnosis. Missing data among alive participants were multiply imputed.

**Results:**

PAM at 9 months was negatively associated with physical and total CRF at both 12 and 24 months after diagnosis, whereas ComQ at 9 months was negatively associated with physical and total CRF at 12 months only. After additional adjustment for previous CRF at 4–6 months, negative associations for PAM with physical and total CRF persisted at 12 months but were attenuated.

**Conclusions:**

Higher patient activation and better perceived patient‐physician communication were associated with lower subsequent CRF, particularly within the first year after diagnosis. Activation‐oriented and communication‐focused strategies may complement existing CRF interventions and warrant further evaluation in pragmatic trials across different survivor groups and settings.

## 1 Background

Cancer‐related fatigue (CRF) is one of the most prevalent and distressing syndromes in cancer survivors during the course of their treatment and recovery [1]. In approximately 25%–30% survivors, CRF persists for years after diagnosis [2, 3]. It is characterized by exhaustion disproportionate to recent activity or rest and may involve multiple symptoms, such as physical lack of energy and weakness, emotional manifestations, including decreased motivation and increased frustration, and cognitive impairments affecting concentration and memory [4, 5, 6]. Despite its high prevalence, impact on daily functioning, and acknowledgment in international guidelines, CRF remains inadequately addressed in cancer care [4, 5, 7]. In Germany, as in many other countries, standard operating procedures regarding CRF are lacking, systematic screening is rare [8], and knowledge among healthcare professionals is insufficient [9, 10, 11, 12]. Consequently, CRF communication is often inadequate [3, 13], leaving patients uninformed about CRF and without referral to evidence‐based interventions such as exercise or psychosocial approaches. These implementation gaps underscore that a sole reliance on acute provider‐driven care structures is insufficient to ensure adequate CRF management. In light of the increasing number of cancer survivors, contemporary survivorship care emphasizes patient‐centered approaches that enable individuals to actively engage in long‐term self‐management of symptoms such as CRF [14, 15]. Within this shared‐care perspective, in which clinician‐ and patient‐driven components are complementary, two modifiable elements may be particularly relevant: patient activation and patient‐physician communication quality.

Patient activation represents an individual's knowledge, skills, and confidence in managing personal health and healthcare [16]. Higher activation levels are associated with adaptive behaviors, better clinical outcomes, and lower healthcare costs in various chronic conditions [17, 18]. Systematic reviews of patient activation in cancer care of various types have documented beneficial associations [19]. Specifically in breast cancer, higher activation has been linked to better health‐related quality of life [18], better treatment adherence [20], and more collaborative relationships with health care professionals [21].

Complementary to patient activation, good patient‐physician communication quality facilitates effective symptom management and survivorship care [22]. To date, communication about CRF is often insufficient. In a recent study, approximately half of participants reported that physicians did not ask about CRF symptoms, and many perceived communication barriers such as uncertainty about whom to contact, limited consultation time, and fear of being perceived as weak or complaining [3]. Prior work further indicates that CRF discussions frequently fail to meet guideline recommendations, which may hinder the development of tailored care [11, 13]. Good patient‐physician communication may reduce anxiety and the feeling of helplessness in patients, improve understanding of CRF management strategies, and foster collaborative care [23, 24]. Thus, enhancing patient activation and patient‐physician communication quality may offer an opportunity to reduce CRF. However, it remains unclear whether cancer survivors who report higher activation and better patient‐physician communication subsequently experience lower levels of CRF. Accordingly, this study investigates whether patient activation and patient‐physician communication quality assessed at 9 months after a cancer diagnosis are associated with subsequent CRF at 12 and 24 months after diagnosis in a German cancer survivor cohort.

## 2 Methods

### 2.1 Study Design and Participants

This analysis utilized data from the “Longitudinal Investigation of cancer‐related Fatigue and its Treatment” (LIFT) project (ClinicalTrials.gov: NCT04921644), a longitudinal, mixed‐methods study investigating the current state of CRF management in Germany from the perspectives of patients, healthcare professionals, and healthcare institutions.

Between August 2021 and September 2022, 1175 cancer patients were recruited for the initial questionnaire. Eligible participants were individuals who were at least 18 years old, possessed the ability to read German, and were capable of following the study protocol. Furthermore, inclusion required a primary tumor diagnosis confirmed by registry data and receipt of active cancer treatment, such as chemotherapy, radiotherapy, hormone therapy, targeted therapy, or immunotherapy. Exclusions comprised secondary carcinomas (except other malignant neoplasms of skin), carcinoma in situ, and inability to provide informed consent. Participants were recruited via the Epidemiological Cancer Registry of Baden‐Württemberg using stratified random sampling by cancer type. Further details are described in another publication from the LIFT project [3].

### 2.2 Measures

#### 2.2.1 Cancer‐Related Fatigue

CRF severity was assessed at 4–6, 9, 12, and 24 months after diagnosis via the validated multidimensional European Organization for Research and Treatment of Cancer Quality of Life Questionnaire Fatigue Module (EORTC QLQ‐FA12). Across 12 items, it assesses physical, emotional, and cognitive CRF symptom subscales, as well as daily life interference and social sequelae on 4‐point Likert scales, referring to symptom experiences during the previous week. Raw scores for each subscale were transformed to 0‐100 scales in accordance with the EORTC scoring manual. Additionally, the present analyses included a total CRF score, utilizing all 12 items together [25]. Higher scores indicate greater CRF severity. According to established thresholds, individual physical, emotional, and cognitive CRF scores above 43, 28, and 25, respectively, are considered clinically relevant [26].

### 2.3 Patient Activation

Patient activation was assessed 9 months after diagnosis via the validated German version of the 13‐item Patient Activation Measure (PAM‐13‐D; subsequently referred to as PAM) [16, 27, 28, 29]. This patient‐reported outcome measure assesses knowledge, skills, and confidence for health self‐management on four‐point Likert scales. Sum scores were transformed into 0‐100 scores, with higher scores indicating greater activation. Missing item responses were imputed according to the PAM scoring guidelines. For the full item list, refer to Supporting Information S1: Appendix Table 1.

### 2.4 Patient‐Physician Communication Quality

Patient‐reported perceptions of patient‐physician communication quality were assessed 9 months after diagnosis via a set of five exploratory items covering clarity of physicians' language, openness to questions and concerns, perceived mutual respect, trust, and overall satisfaction with communication during medical encounters (subsequently referred to as ComQ). Responses were provided on four‐point Likert scales and combined into a sum score that was linearly transformed to a 0–100 communication score, with higher values indicating better perceived patient‐physician communication. For the item “My physicians often use terms that I do not understand”, response valence was reversed so that lower original scores corresponded to higher ComQ values in line with the other items. Internal consistency of the five items was good (Cronbach's *α* = 0.84, *n* = 950). Where required, single missing items were imputed by replacing the missing value with the individual's mean of the remaining items, but only if exactly one of the five items was missing. For the full item list, refer to Supporting Information S1: Appendix Table 1. As this study‐specific ComQ scale has not undergone formal psychometric validation, its findings should be interpreted as exploratory.

### 2.5 Covariates

Covariates were included to account for potential confounding of the association between the independent variables, PAM and ComQ, and the outcome, CRF scores, based on subject‐matter knowledge and prior literature. Sociodemographic covariates included sex (female vs. male), age at diagnosis (continuous in years), educational level, and living situation. Educational level was dichotomized into high versus low education. High education included a high school degree (German “(Fach‐)Abitur”), a completed university or applied‐sciences degree, or a master craftsman qualification (German “Meisterprüfung”). All other qualifications were categorized as low education. Living situation was coded as living alone versus living with others. All sociodemographic data were self‐reported 4–6 months after diagnosis.

Clinical covariates included cancer stage at diagnosis (I‐II, III, IV, X/uncertain), metastases 4–6 months after diagnosis (yes/no), number of comorbidities, and current cancer treatment status to control for disease severity and overall clinical burden. Comorbidity was operationalized as a comorbidity count based on patient self‐report 4–6 months after diagnosis, excluding psychiatric conditions to avoid overadjustment given their strong correlation with CRF.

To control for the influence of cancer treatment on CRF, we considered four major treatment modalities: chemotherapy, radiotherapy, hormone therapy, and targeted/immunotherapy. For each modality, treatment status was categorized as never, current or recently completed within the previous 6 months, completed over 6 months ago, or unknown. Data on treatment modalities were available at every timepoint except 9 months.

### 2.6 Statistical Analysis

Sample characteristics of the 9‐month sample, corresponding to the timepoint at which both PAM and ComQ were assessed, were described. Continuous variables were described using means, standard deviations, medians and quartiles. Categorical variables were described using absolute and relative frequencies. Moreover, single‐item distributions of PAM and ComQ were visualized to assess item‐level differences.

The primary analyses were multiple linear regression models examining associations of PAM and ComQ assessed 9 months after diagnosis with CRF 12 and 24 months after diagnosis. Separate models were performed for physical, emotional, cognitive, and total CRF as dependent variables. Based on the theory‐led assumption for possible confounding, all models were adjusted for sociodemographic covariates (sex, age, educational level, living status), markers of disease severity and clinical burden (cancer stage, metastases, number of comorbidities), and current treatment status for all treatment modalities mentioned above.

Additionally, potential bidirectional relationships between CRF, patient activation, and patient‐physician communication quality were considered. Earlier CRF may influence how patients report activation and communication, but each of them may also affect subsequent CRF. To address this, additional sensitivity models with adjustment for earlier CRF (four to six months after diagnosis) were run, assuming that the true effects lie between the estimates from the primary and sensitivity models.

Missing data at follow‐up, mainly due to missed assessments or dropout from the study, were assumed to be driven by a poor or worsening health status, and hence likely not missing completely at random. Therefore, restricting analyses to complete cases could be biased. Thus, missing outcome and covariate data for participants who were alive at follow‐up were handled using multiple imputation. Specifically, 20 imputed datasets including all analysis variables were generated via the SPSS embedded fully conditional specification algorithm. Estimates from each dataset were pooled via application of Rubin's rules.

Regression assumptions were fulfilled in the models assessing the physical and total CRF subscales. For emotional and cognitive CRF, the normality of residuals assumption was violated, likely due to zero inflation. Thus, these subscales are only reported descriptively, and no regression analyses are presented to avoid potentially misleading estimates from poorly fitting models. All analyses were performed with SPSS 30 (IBM).

## 3 Results

### 3.1 Sample Characteristics

**FIGURE 1:**
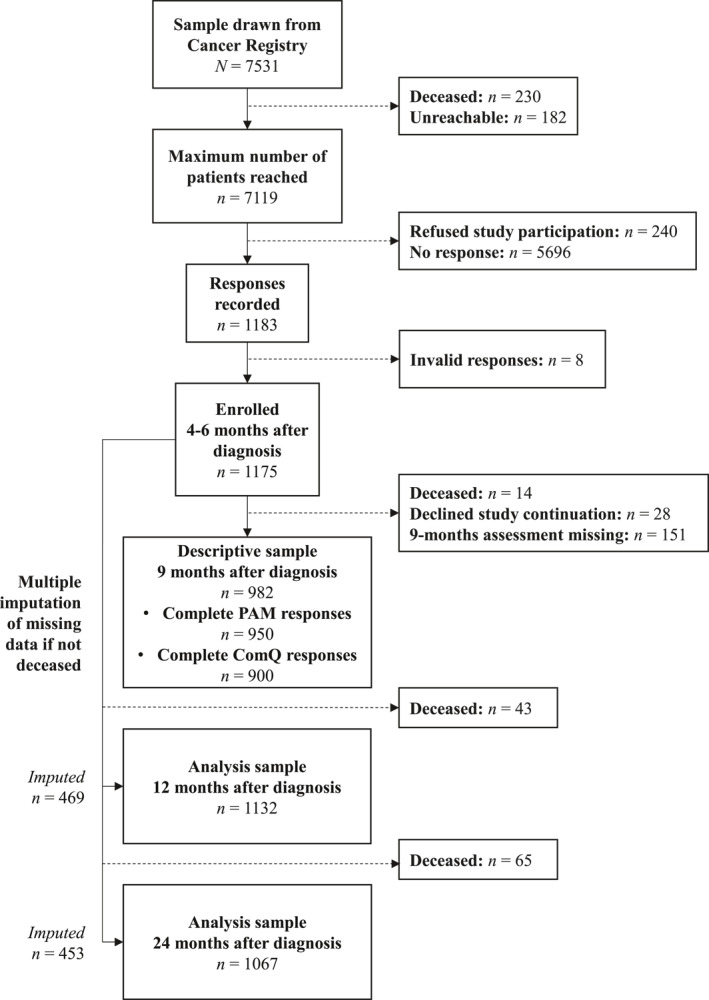
Sample flow. CRF was assessed at 4–6, 9, 12, and 24 months after diagnosis; PAM and ComQ were assessed at 9 months only. The numbers shown in the boxes up to 9 months represent observed questionnaire data. For the regression analyses, missing predictor, outcome and covariate data among participants were handled using multiple imputation, unless patients were deceased. Specifically, 20 imputations were performed within the full cohort of participants who completed the 4–6‐month questionnaire (*n* = 1175), using information from all available timepoints and variables. ComQ = patient‐physician communication quality; CRF = cancer‐related fatigue; PAM = Patient Activation Measure.

The sample flow is presented in Figure 1. Sample characteristics at 9 months after diagnosis, corresponding to the assessment of PAM and ComQ, are presented in Table 1. The 9‐month sample (*n* = 982) comprised 55% female participants, a mean age in the early sixties at diagnosis, and predominantly low educational level (61%). Breast cancer was the most frequent diagnosis (28%), followed by colorectal (16%) and prostate cancer (15%), with remaining participants distributed across lung cancer, melanoma, hematologic malignancies, gynecological tumors, and other sites. Most patients were in stages I or II at diagnosis (54%), had no documented metastases 4–6 months after diagnosis (78%), and the median number of comorbidities was 2 Q1; Q3 = [1, 3].

**TABLE 1:** Patient characteristics and cancer‐related fatigue (CRF) 9 months after diagnosis, stratified by patient activation (PAM) patient‐physician communication quality (ComQ).

	Total (*n* = 982)		Low PAM (*n* = 148)		High PAM (*n* = 802)		Low ComQ (*n* = 280)		High ComQ (*n* = 620)	
	Mean	SD	Mean	SD	Mean	SD	Mean	SD	Mean	SD
	*n*	%	*n*	%	*n*	%	*n*	%	*n*	%
Age at diagnosis [years]	63.38	12.34	63.50	12.50	63.35	12.34	64.64	11.04	61.79	12.77
Sex										
Female	544	55.4	75	50.7	446	55.6	140	50.0	353	56.9
Male	438	44.6	73	49.3	356	44.4	140	50.0	267	43.1
Educational level^a^										
Low	594	61.2	101	70.1	471	59.2	180	65.7	353	57.4
High	377	38.8	43	29.9	324	40.8	94	34.3	262	42.6
Living situation										
With others	802	82.2	114	78.6	660	82.6	217	78.1	526	85.3
Alone	174	17.8	31	21.4	139	17.4	61	21.9	91	14.7
Cancer type										
Breast	273	27.9	27	18.2	237	29.7	54	19.4	198	32.0
Colorectal	161	16.4	30	20.3	125	15.6	54	19.4	93	15.0
Prostate	142	14.5	16	10.8	124	15.5	41	14.7	91	14.7
Lung	71	7.3	13	8.8	55	6.9	21	7.6	38	6.1
Melanoma	62	6.3	12	8.1	49	6.1	21	7.6	36	5.8
Lymphoma	43	4.4	6	4.1	37	4.6	14	5.0	29	4.7
Multiple localizations^b^	38	3.9	6	4.1	30	3.8	17	6.1	20	3.2
Pancreas	31	3.2	7	4.7	23	2.9	8	2.9	20	3.2
Leukemia	31	3.2	4	2.7	25	3.1	4	1.4	23	3.7
Stomach	28	2.9	7	4.7	20	2.5	7	2.5	17	2.7
Thyroid gland	28	2.9	6	4.1	18	2.3	9	3.2	19	3.1
Gynecological tumors	28	2.9	4	2.7	23	2.9	9	3.2	15	2.5
Bladder or kidney	25	2.5	4	2.7	21	2.6	11	3.9	10	1.6
Liver	18	1.8	6	4.1	12	1.5	8	2.9	10	1.6
Stage at diagnosis										
I‐II	491	54.4	57	41.3	418	56.9	129	49.8	322	56.9
III	129	14.3	23	16.7	101	13.7	41	15.8	72	12.7
IV	144	15.9	27	19.6	114	15.5	45	17.4	91	16.1
X	139	15.4	31	22.5	102	13.9	44	17.9	81	14.3
Metastasis^c^										
No	770	78.4	104	70.3	639	79.7	215	76.8	429	79.4
Yes	182	18.5	39	26.4	139	17.3	50	17.9	114	18.4
Unknown	30	3.1	5	3.4	24	3.0	15	5.4	14	2.3
Chemotherapy^c^										
Never	371	37.8	54	36.5	304	37.9	118	42.1	232	37.4
Current or recently completed	396	40.3	69	46.6	318	39.7	108	38.6	259	41.8
Completed over 6 months ago	19	1.9	4	2.7	15	1.9	6	2.1	8	1.3
Unknown	196	20.0	21	14.2	165	20.6	48	17.1	121	19.5
Radiotherapy^c^										
Never	394	40.1	55	37.2	323	40.3	117	41.8	253	40.8
Current or recently completed	293	29.8	38	25.7	246	30.7	82	29.3	190	30.6
Completed over 6 months ago	43	4.4	7	4.7	35	4.4	14	5.0	26	4.2
Unknown	252	25.7	48	32.4	198	24.7	67	23.9	151	24.4
Hormone therapy^c^										
Never	397	40.4	53	35.8	329	41.0	116	41.4	258	41.6
Current or recently completed	227	23.1	29	19.6	192	23.9	55	19.6	159	25.6
Completed over 6 months ago	1	0.1	1	0.7	0	0.0	1	0.4	0	0.0
Unknown	357	36.4	65	43.9	281	35.0	108	38.6	203	32.7
Targeted/immunotherapy^c^										
Never	445	45.3	60	40.5	370	46.1	131	46.8	291	46.9
Current or recently completed	191	19.5	35	23.6	150	18.7	47	16.8	132	21.3
Completed over 6 months ago	3	0.3	1	0.7	2	0.2	1	0.4	2	0.3
Unknown	343	34.9	52	35.1	280	34.9	101	36.1	195	31.5

	Median	Q1; Q3	Median	Q1; Q3	Median	Q1; Q3	Median	Q1; Q3	Median	Q1; Q3
Comorbidity count	2	1; 3	2	1; 4	2	1; 3	2	1; 3	1	0; 2
CRF 9 months after diagnosis^d^										
Physical CRF	47	27; 67	67	47; 82	47	27; 67	60	38; 73	43	27; 67
Emotional CRF^e^	22	0; 44	44	17; 67	11	0; 44	33	11; 55	11	0; 44
Cognitive CRF^e^	17	0; 33	17	0; 50	17	0; 33	17	0; 33	17	0; 33
Total CRF	33	17; 52	47	33; 69	31	14; 47	42	25; 61	31	14; 47
PAM^d^	72	61; 79	48	41; 51	72	67; 82	64	56; 77	72	64; 82
ComQ^d^	73	60; 93	67	47; 73	73	60; 93	53	33; 60	87	73; 93

*Note:* Due to missing values, frequencies do not always add up to total. Percentages are based on observed data (valid cases). Because the patient‐physician communication quality scale has more missing data than the Patient activation scale, the communication‐stratified sample is smaller than the activation‐stratified sample. PAM was dichotomized using the midpoint of the four validated PAM levels (low activation: levels 1–2; high activation: levels 3–4). ComQ mean scores range from 1–4; low communication quality: mean < 3; high communication quality: mean ≥ 3.

^a^ *Low*: no degree or lower secondary education degree; *High:* diploma qualifying for university/university degree, or master craftsman qualification.

^b^ Multiple primary tumors at different locations.

^c^ Based on registry data and self‐reports 4–6 months after diagnosis.

^d^ Assessed 9 months after diagnosis.

^e^ Emotional and cognitive CRF subscales are presented descriptively only; they were not included in the regression models due to violation of model assumptions.

Patients with low PAM were more likely to have low educational levels than those with high PAM (70% vs. 59%) and were slightly more likely to live alone (21% vs. 17%). Median physical and total CRF scores at 9 months were higher in the low‐PAM group than in the high‐PAM group (Median (Q1, Q3) for physical: 67 (47; 82) versus 47 (27; 67); total: 47 (33; 69) versus 31 (14; 47)). For emotional CRF, the low‐PAM group showed higher median scores than the high‐PAM group (44 (17; 67) versus 11 (0; 44)), while for cognitive CRF, both groups showed a less pronounced difference (17 (0; 50) versus 17 (0; 33)).

Comparisons by ComQ showed that patients reporting low communication quality tended to be slightly older at diagnosis than those reporting high communication quality (mean = 65 vs. 62 years), more often had low education (66% vs. 58%) and lived alone (22% vs. 15%). Median physical and total CRF scores were higher in the low‐ComQ group compared with the high‐ComQ group (physical: 60 (38; 73) versus 43 (27; 67); total: 42 (25; 61) versus 31 (14; 47)). Emotional CRF was higher in the low‐ComQ group (33 (11; 55)) than in the high‐ComQ group (11 (0; 44)), while median cognitive CRF scores were similar across communication groups (17 (0; 33) in both).

### 3.2 Perceived Patient Activation and Patient‐Physician Communication Quality

**FIGURE 2:**
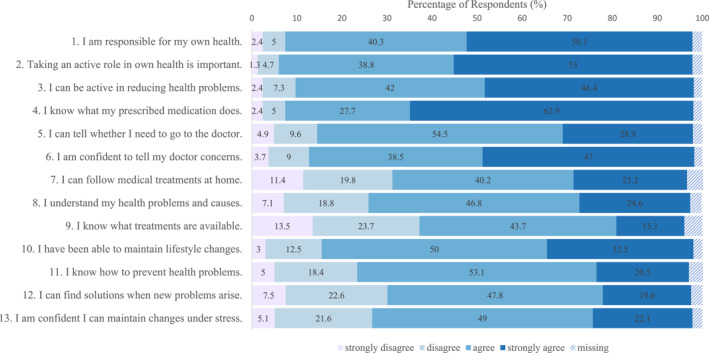
Item‐level distribution of responses to the Patient Activation Measure. Distributions are based on observed data 9 months after diagnosis (*n* = 982). For clearer presentation, item phrasing was abbreviated (e.g., “Taking an active role in own health is important.” in place of the full phrasing “Taking an active role in my own health care is the most important thing that affects my health.“). For the full item phrasing, see Appendix Table 1.

Distributions of single‐item responses of PAM, assessed 9 months after diagnosis, are presented in Figure 2. For all 13 PAM items, response distributions were skewed toward agreement, indicating generally high patient activation in this sample. For earlier PAM items reflecting more basic beliefs and confidence about one's role in managing health (items 1‐6, e.g., “1. I am responsible for my own health.“, “2. Taking an active role in own health is important.“), 86%–93% of participants selected “agree” or “agree strongly”. In contrast, later items targeting more concrete self‐management tasks and capabilities (items 7–13, e.g., “7. I can follow medical treatments at home.“, “13. I am confident I can maintain changes under stress.“) showed greater heterogeneity with 14%–30% of participants selecting “disagree” or “disagree strongly”.

**FIGURE 3:**
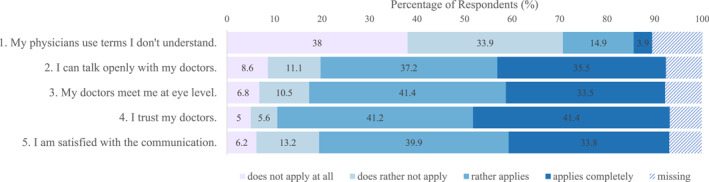
Item‐level distribution of responses to items assessing patient‐physician communication quality. Distributions are based on observed data 9 months after diagnosis (*n* = 982). For clearer presentation, item phrasing was abbreviated (e.g., I can talk openly with my doctors. in place of the full phrasing I can discuss my questions and concerns openly with my doctors.). For the full item phrasing, see Supporting Information S1: Appendix Table 1.

Distributions of responses of the five ComQ items (Figure 3) also indicated predominantly positive perceptions of communication with physicians. However, between 11% and 22% of patients reported unsatisfactory communication aspects, such as physicians using incomprehensible language, inability to talk openly and at eye level with the physician, and general dissatisfaction with the communication.

### 3.3 Associations of Patient Activation and Patient‐Physician Communication Quality With Cancer‐Related Fatigue

Pooled results of the primary multiple linear regression analyses based on multiply imputed data are summarized in Table 2A. PAM scores at 9 months were negatively associated with physical CRF at both 12 and 24 months (12 months: *B* = −0.30, 95% CI [−0.40; −0.19], *p* < 0.001; 24 months: *B* = −0.23, 95% CI [−0.34; −0.12], *p* < 0.001). ComQ scores were also negatively associated with physical CRF at 12 months (*B* = −0.11, 95% CI [−0.18; −0.03], *p* = 0.005), whereas the association with physical CRF at 24 months was small and not statistically significant. For total CRF, PAM scores were likewise negatively associated with CRF at both follow‐ups (12 months: *B* = −0.28, 95% CI [−0.37; −0.20], *p* < 0.001; 24 months: *B* = −0.21, 95% CI [−0.30; −0.12], *p* < 0.001). ComQ scores were negatively associated with total CRF at 12 months (*B* = −0.10, 95% CI [−0.16; −0.04], *p* = 0.002), while the association at 24 months was weaker and narrowly missed conventional statistical significance (*B* = −0.06, 95% CI [−0.12; 0.00], *p* = 0.060).

**TABLE 2:** Associations of patient activation (PAM) and patient‐physician communication quality (ComQ) with physical and total cancer‐related fatigue (CRF), using multiply imputed data.

Variable	12‐Month Physical Fatigue			24‐Month Physical Fatigue		
	B	95% CI	*p*	B	95% CI	*p*
A. Primary Analyses						
PAM	**−0.30**	(−0.40; −0.19)	**< 0.001**	**−0.23**	(−0.34; −0.12)	**< 0.001**
ComQ	**−0.11**	(−0.18; −0.03)	**0.005**	−0.04	(0.13; 0.04)	0.281

	12‐Month Total fatigue			24‐Month Total fatigue		
	B	95% CI	*p*	B	95% CI	*p*
PAM	**−0.28**	(−0.37; −0.20)	**< 0.001**	**−0.21**	(−0.30; −0.12)	**< 0.001**
ComQ	**−0.10**	(−0.16; −0.04)	**0.002**	−0.06	(−0.12; 0.00)	0.060

Variable	12‐Month Physical Fatigue			24‐Month Physical Fatigue		
	B	95% CI	*p*	B	95% CI	*p*
B. Sensitivity Analyses						
PAM	**−0.12**	(−0.22; −0.03)	**0.014**	−0.08	(−0.18; 0.03)	0.151
ComQ	**−0.07**	(−0.15; −0.00)	**0.041**	−0.02	(−0.09; 0.06)	0.688
Previous physical CRF	**0.45**	(0.39; 0.50)	**< 0.001**	**0.40**	(0.34; 0.45)	**< 0.001**

	12‐Month Total Fatigue			24‐Month Total Fatigue		
	B	95% CI	*p*	B	95% CI	*p*
PAM	**−0.11**	(−0.19; −0.03)	**0.005**	−0.06	(−0.15; 0.02)	0.149
ComQ	−0.05	(−0.10; 0.01)	0.091	−0.02	(−0.07; 0.04)	0.578
Previous CRF	**0.48**	(0.43; 0.53)	**< 0.001**	**0.42**	(0.37; 0.47)	**< 0.001**

*Note:* All results are pooled estimates of 20 multiply imputed datasets (*n* = 1132 at 12 months; *n* = 1067 at 24 months). If a participant was deceased, missing values after death were not imputed. All models are adjusted for age at diagnosis, sex (female/male), education (low/high), living situation (with others/alone), number of comorbidities 4–6 months after diagnosis, cancer stage (I‐II, III, IV, X), metastases 4–6 months after diagnosis (yes/no), and current status of chemo‐, radio‐, hormone‐, and targeted/immunotherapy at the respective 12 or 24 months follow‐up (never, current or recently completed, completed > 6 months ago, unknown). The sensitivity analyses further adjust for previous fatigue 4–6 months after diagnosis. Effect estimates are unstandardized regression coefficients. PAM, ComQ and previous CRF are all 0‐100 scales.

Sensitivity analyses additionally adjusting for previous CRF (4–6 months after diagnosis) are presented in Table 2B. PAM continued to show negative, but attenuated associations with physical and total CRF 12 months after diagnosis (physical CRF *B*‐PAM = −0.12, 95% CI [−0.21; −0.03], *p* = 0.012; total CRF: *B* = −0.11, 95% CI [−0.19; −0.03], *p* = 0.005). The association of ComQ with physical CRF at 12 months was also attenuated (*B* = −0.07, 95% CI [−0.15; −0.00], *p* = 0.041), but remained statistically significant, whereas the association with total CRF did not reach significance. For CRF at 24 months after diagnosis, the associations of PAM and ComQ with physical and total CRF were further attenuated and were not statistically significant. Previous CRF was strongly and positively associated with subsequent physical and total CRF at both time points (12 months: *B* = 0.45–0.48 and 24 months: *B* = 0.40–0.42; all *p* < 0.001).

The estimates from multiple‐imputed datasets did not differ substantially from those based on complete cases. Further exploratory sensitivity analyses were conducted to clarify whether associations were driven by specific subgroups. Restricting analyses to participants without metastases 4–6 months after diagnosis yielded results comparable to the main models (Supporting Information S1: Appendix Table 2). Similarly, sex‐stratified models indicated broadly comparable associations across men and women (Supporting Information S1: Appendix Tables 3 and 4).

## 4 Discussion

Patients who reported higher patient activation and better patient‐physician communication quality 9 months after diagnosis tended to report lower physical and total CRF at 12 months, with higher activation also being associated with lower physical and total CRF at 24 months in the primary analyses. After additional adjustment for previous CRF at 4–6 months, these associations at 12 months largely persisted but were attenuated, whereas associations at 24 months became weaker and were no longer statistically significant. The results suggest that patient activation and patient‐physician communication quality during the acute‐to‐recovery phase may be relevant for CRF severity within the first year after diagnosis, with activation potentially being relevant for longer‐term CRF as well, while any contribution of early communication on longer‐term CRF may be overshadowed by other, later‐occurring factors.

Although overall patient activation levels were high in this sample, consistent with other oncology research [19, 29, 30], item‐level analyses revealed gaps in higher‐level activation domains. Items capturing general responsibility and confidence showed ceiling effects and mainly reflected that most survivors endorsed core beliefs about being proactive in their own health. In contrast, more demanding items revealed difficulties in roughly one fifth to one third of respondents with sustained self‐management, including maintaining lifestyle changes, knowing how to prevent future health problems, problem‐solving in new situations, and maintaining healthy behaviors under stress. Since CRF management requires not only initial behavior adoption but also maintenance despite competing stressors, and adaptive problem‐solving when symptoms fluctuate [6, 31], these difficulties seem specifically relevant. Given these item‐level gaps and the observed negative associations of PAM with CRF, activation‐oriented approaches targeting these specific domains may offer a promising avenue.

Item‐level distributions on the ComQ scale indicated generally favorable communication experiences, though 11%–22% reported unsatisfactory aspects, including feeling less involved, less trusting, being hindered by medical jargon, or generally being dissatisfied with communication. The negative association between ComQ and CRF at 12 months underlines the potential of providing understandable, empathetic CRF communication that encourages patients to voice concerns. This aligns with identified care gaps: physicians often lack guideline‐based knowledge about CRF and rarely initiate such discussions, patients receive limited education [9], and two‐thirds of cancer survivors perceive barriers to discussing CRF [3]. Additionally, interprofessional uncertainty about roles and responsibilities in CRF management creates structural barriers [13, 32]. The present findings suggest that both patient‐level and system‐level improvements in CRF‐related communication may be beneficial.

## 5 Implications (Clinical and Research)

Considering the multifactorial nature of CRF, the typically small‐to‐moderate effects of single interventions [33, 34], and the modest associations from the present analyses, activation‐based and communication‐facilitating strategies should be conceptualized as complementary components within broader, multimodal CRF care pathways. Interventions that strengthen patients' skills and confidence for day‐to‐day self‐management may contribute to additional, incremental reductions in CRF, particularly when embedded into established CRF interventions (e.g., exercise programs or psychoeducational support). A stratified approach tailoring intensity to patients' activation profiles may be beneficial [35]. For survivors with low activation, brief, structured support breaking self‐management tasks into small, attainable steps may reduce feelings of overload. For example, patients might start with short walks and gradually increase duration and frequency in the context of facilitating physical activity [3, 36]. For survivors who accept general responsibility but struggle to maintain health behaviors, peer‐led programs delivered by trained cancer survivors could normalize setbacks, enhance self‐efficacy, and provide strategies for integrating activity into everyday life, as such models have shown promise in improving self‐management skills through motivational interviewing and shared experiential learning [37]. Such approaches may be particularly relevant for survivors with limited day‐to‐day social support, including those living alone, who may face additional challenges in maintaining health behaviors and navigating CRF‐related care.

The negative associations between ComQ and CRF indicate communication processes as another plausible target for CRF management. Several communication mechanisms appear actionable. First, clarifying professional roles and referral pathways for CRF screening, education, and follow‐up within multidisciplinary teams may reduce interprofessional ambiguity that currently hinders CRF‐related conversations [13, 32]. Second, explicitly framing CRF as a legitimate and expected consequence of cancer and its treatment may counter patients' communication barriers and help clinicians address a symptom that can seem less tangible than other disease outcomes [6, 9, 38]. Third, presenting CRF management as a long‐term process, setting realistic, incremental goals, and consistently using plain, jargon‐free language when explaining evidence‐based strategies, may foster shared understanding and reduce the risk that both patients and healthcare professionals feel overwhelmed [6, 39].

Overall, these implications suggest that brief, low‐intensity interventions combining tailored activation support with improved CRF‐related communication could be integrated as scalable components of routine care during the first year after diagnosis, when symptom‐management routines are being established and when these factors appear most closely linked to CRF [1, 3]. Even modest individual effects might be meaningful if implemented widely alongside established CRF interventions such as exercise and psychoeducation [6]. By jointly modeling patient activation and patient‐physician communication, this study adds a patient‐centered perspective on modifiable factors in CRF management warranting further investigation.

Prospective studies with repeated assessments of activation, communication quality, and CRF are needed to clarify the temporal dynamics and potential causal pathways. Pragmatic trials evaluating brief, stratified activation‐oriented interventions embedded in routine survivorship care, particularly during the first year after diagnosis, would help establish whether the targets identified here translate into meaningful CRF reductions. Future research should also examine whether CRF communication training for healthcare professionals improves not only patients' perceived communication quality but also downstream CRF outcomes.

### 5.1 Study Limitations

First, the study relied on patient‐reported outcome measures, as no objective measurements for patient activation, patient‐physician communication, or CRF exist. This may introduce response biases, including social desirability in reporting activation, particularly for PAM items that measure more general aspects of activation. Higher activation may also be associated with protective personality traits and coping mechanisms that have not been accounted for in the analyses. The study‐specific ComQ scale, although showing good internal consistency, has not undergone formal psychometric validation.

Moreover, the observational design limits causal inference despite the temporal ordering. Bidirectional relationships between CRF, patient activation, and communication quality at earlier phases of survivorship may contribute to residual confounding that cannot be fully resolved even by our sensitivity analyses.

Another limitation is that PAM and ComQ were each assessed only once, at 9 months after diagnosis, whereas CRF was measured repeatedly over time. This limits insight into potential changes in patient activation and perceived communication quality during survivorship and how these relate to CRF. Furthermore, selective attrition likely affected sample composition over time due to illness‐related dropout, loss to follow‐up, or mortality [40]. Although multiple imputations were applied using a comprehensive set of covariates and the outcome variables to reduce bias, some residual bias may remain, as participants who complete all assessments tend to have better performance status and fewer symptoms than those who drop out.

Finally, external validity is limited by the response proportion of the registry‐based sampling, which raises the possibility of selection bias, and by the fact that the final sample was not fully representative of the underlying German cancer population. In addition, some cancer types were represented by relatively small subgroups. These factors may limit generalizability of the findings. The sample was also characterized by relatively high activation scores, which may have restricted variability and attenuated observed associations, potentially leading to conservative effect estimates.

Strengths of this study are its large, registry‐based sample, the longitudinal design with repeated CRF assessments, and the use of validated multidimensional CRF and activation measures. Furthermore, by jointly modeling patient activation and patient‐physician communication, it provides a patient‐centered perspective on modifiable factors that may be relevant for CRF management during the acute‐to‐recovery phase and help to inform the design of future supportive care interventions.

## 6 Conclusion

This longitudinal study showed that higher patient activation and better perceived patient‐physician communication during the acute‐to‐recovery phase were associated with lower subsequent physical and total CRF, particularly within the first year after diagnosis. While these associations attenuated over time, the findings underline that modifiable, patient‐centered resources may help shape CRF trajectories alongside factors such as sociodemographic and clinical characteristics. These results support integrating activation‐oriented and communication‐focused strategies as low‐cost, modifiable leverage points into routine CRF care, where they should complement rather than replace comprehensive multidisciplinary assessment and interventions.

## Author Contributions

Conceptualization: M.S., I.M., K.S. Data curation: M.M., A.W., S.K. Formal analysis: D.T.N., M.S. Writing–original draft: D.T.N., M.S. Writing–review and editing: D.T.N., M.S., M.M., A.W., S.K., I.M., K.S. Supervision: M.S., I.M., K.S. Funding acquisition: M.S., I.M., K.S.

## Ethics Statement

The study was approved by the Ethics Committee of the Medical Faculty of Heidelberg University (S‐526/2018) and fully complied with the Declaration of Helsinki.

## Conflicts of Interest

The authors declare no conflicts of interest.

## Supporting information


Supporting Information S1

## Data Availability

Data can be made available to scientific cooperation partners upon reasonable request.
