# Supplementary material for: Patient Activation, Patient‐Physician Communication Quality, and Cancer‐Related Fatigue: A Longitudinal Study of Cancer Survivors
Source: Psychooncology. 2026 Jul 14;35(7):e70542. doi: 10.1002/pon.70542 (PMC13370050; doi:10.1002/pon.70542)
Supplement: Supplementary file 1 — Supporting Information S1 [file PON-35-e70542-s001.docx]

**Appendix Table 1. Full item phrasing of the 13‑item Patient Activation Measure (PAM‑13; PAM) and the exploratory assessment of patient-physician communication quality (ComQ).**

| Item | Phrasing |
| --- | --- |
| PAM |  |
| 1 | When all is said and done, I am the person who is responsible for taking care of my health. |
| 2 | Taking an active role in my own health care is the most important thing that affects my health. |
| 3 | I am confident I can help prevent or reduce problems associated with my health. |
| 4 | I know what each of my prescribed medications do. |
| 5 | I am confident that I can tell whether I need to go to the doctor or whether I can take care of a health problem myself. |
| 6 | I am confident that I can tell a doctor concerns I have even when he or she does not ask. |
| 7 | I am confident that I can follow through on medical treatments I may need to do at home. |
| 8 | I understand my health problems and what causes them. |
| 9 | I know what treatments are available for my health problems. |
| 10 | I have been able to maintain (keep up with) lifestyle changes, like eating right or exercising. |
| 11 | I know how to prevent problems with my health. |
| 12 | I am confident I can figure out solutions when new problems arise with my health. |
| 13 | I am confident that I can maintain lifestyle changes, like eating right and exercising, even during times of stress. |
| ComQ |  |
| 1 | My physicians often use terms that I do not understand. |
| 2 | I can discuss my questions and concerns openly with my doctors. |
| 3 | My doctors meet me at eye level. |
| 4 | I trust my doctors. |
| 5 | I am satisfied with the communication with my doctors. |

*Note.
PAM items shown are the original English PAM‑13 phrasings by Hibbard et al. (2004), corresponding to the German version (PAM‑13‑D) by Brenk‑Franz et al. (2013) used in this study.*

**Appendix Table 2. Associations of patient activation (PAM) and patient-physician communication quality (ComQ) with physical and total cancer-related fatigue (CRF), using multiply imputed data of participants without metastasis**

***A. Primary Analyses***

| Variable | 12-Month Physical Fatigue | | | 24-Month Physical Fatigue | | |
| --- | --- | --- | --- | --- | --- | --- |
|  | B | 95% CI | *p* | B | 95% CI | *p* |
| PAM | **-.26** | (-.38; -.15) | **<.001** | **-.22** | (-.34; -.09) | **<.001** |
| ComQ | **-.11** | (-.20; -.03) | **.008** | -.04 | (-.12; .04) | .358 |
|  | 12-Month Total Fatigue | | | 24-Month Total Fatigue | | |
|  | B | 95% CI | *p* | B | 95% CI | *p* |
| PAM | **-.26** | (-.36; -.17) | **<.001** | **-.21** | (-.31; -.11) | **<.001** |
| ComQ | **-.09** | (-.16; -.03) | **.006** | -.05 | (-.11; .02) | .176 |

***B. Sensitivity Analyses***

| Variable | 12-Month Physical Fatigue | | | 24-Month Physical Fatigue | | |
| --- | --- | --- | --- | --- | --- | --- |
|  | B | 95% CI | *p* | B | 95% CI | *p* |
| PAM | -.10 | (-.21; .00) | .070 | -.07 | (-.18; .05) | .261 |
| ComQ | **-.08** | (-.16; -.01) | **.032** | -.01 | (-.09; .07) | .688 |
| Previous physical CRF | **.45** | (.39; .50) | **<.001** | **.41** | (.35; .47) | **<.001** |
|  | 12-Month Total Fatigue | | | 24-Month Total Fatigue | | |
|  | B | 95% CI | *p* | B | 95% CI | *p* |
| PAM | **-.10** | (-.19; -.02) | **.020** | -.08 | (-.17; .02) | .111 |
| ComQ | -.05 | (-.11; .01) | .121 | -.01 | (-.07; .05) | .823 |
| Previous total CRF | **.48** | (.43; .54) | **<.001** | **.42** | (.37; .48) | **<.001** |

*Note.
All results are pooled estimates of 20 multiply imputed datasets ( n = 935 at 12 months; n = 904 at 24 months). If a participant was deceased, missing values after death were not imputed. All models are adjusted for age at diagnosis, sex (female/male), education (low/high), living situation (with others/alone), number of comorbidities 4-6 months after diagnosis, cancer stage (I-II, III, IV, X), metastases 4-6 months after diagnosis (yes/no), and current status of chemo-, radio-, hormone-, and targeted/immunotherapy at the respective 12 or 24 months follow-up (never, current or recently completed, completed >6 months ago, unknown). The sensitivity analyses further adjust for previous fatigue 4-6 months after diagnosis. Effect estimates are unstandardized regression coefficients. PAM, ComQ and previous CRF are all 0-100 scales.*

**Appendix Table 3. Associations of patient activation (PAM) and patient-physician communication quality (ComQ) with physical and total cancer-related fatigue (CRF), using multiply imputed data of male participants**

***A. Primary Analyses***

| Variable | 12-Month Physical Fatigue | | | 24-Month Physical Fatigue | | |
| --- | --- | --- | --- | --- | --- | --- |
|  | B | 95% CI | *p* | B | 95% CI | *p* |
| PAM | **-.34** | (-.50; -.18) | **<.001** | **-.22** | (-.38; -.06) | **.006** |
| ComQ | -.08 | (-.20; .03) | .154 | -.08 | (-.18; .03) | .171 |
|  | 12-Month Total Fatigue | | | 24-Month Total Fatigue | | |
|  | B | 95% CI | *P* | B | 95% CI | *p* |
| PAM | **-.32** | (-.45; -.19) | **<.001** | **-.19** | (-.31; -.06) | **.003** |
| ComQ | -.09 | (-.17; .00) | .058 | -.08 | (-.16; .01) | .077 |

***B. Sensitivity Analyses***

| Variable | 12-Month Physical Fatigue | | | 24-Month Physical Fatigue | | |
| --- | --- | --- | --- | --- | --- | --- |
|  | B | 95% CI | *P* | B | 95% CI | *p* |
| PAM | -.10 | (-.24; .05) | .186 | -.06 | (-.21; .09) | .435 |
| ComQ | -.05 | (-.15; .06) | .409 | -.05 | (-.15; .07) | .688 |
| Previous physical CRF | **.50** | (.42; .58) | **<.001** | **.35** | (.27; .44) | **<.001** |
|  | 12-Month Total Fatigue | | | 24-Month Total Fatigue | | |
|  | B | 95% CI | *p* | B | 95% CI | *p* |
| PAM | -.09 | (-.20; .03) | .143 | -.03 | (-.14; .09) | .679 |
| ComQ | -.03 | (-.11; .05) | .501 | -.04 | (-.11; .04) | .377 |
| Previous total CRF | **.40** | (.46; .61) | **<.001** | **.42** | (.32; .48) | **<.001** |

*Note.
All results are pooled estimates of 20 multiply imputed datasets ( n = 499 at 12 months; n = 461 at 24 months). If a participant was deceased, missing values after death were not imputed. All models are adjusted for age at diagnosis, sex (female/male), education (low/high), living situation (with others/alone), number of comorbidities 4-6 months after diagnosis, cancer stage (I-II, III, IV, X), metastases 4-6 months after diagnosis (yes/no), and current status of chemo-, radio-, hormone-, and targeted/immunotherapy at the respective 12 or 24 months follow-up (never, current or recently completed, completed >6 months ago, unknown). The sensitivity analyses further adjust for previous fatigue 4-6 months after diagnosis. Effect estimates are unstandardized regression coefficients. PAM, ComQ and previous CRF are all 0-100 scales.*

**Appendix Table 4. Associations of patient activation (PAM) and patient-physician communication quality (ComQ) with physical and total cancer-related fatigue (CRF), using multiply imputed data of female participants.**

***A. Primary Analyses***

| Variable | 12-Month Physical Fatigue | | | 24-Month Physical Fatigue | | |
| --- | --- | --- | --- | --- | --- | --- |
|  | B | 95% CI | *p* | B | 95% CI | *p* |
| PAM | **-.27** | (-.41; -.13) | **<.001** | **-.24** | (-.39; -.09) | **.001** |
| ComQ | **-.13** | (-.23; -.02) | **.023** | -.02 | (-.14; .09) | .678 |
|  | 12-Month Total Fatigue | | | 24-Month Total Fatigue | | |
|  | B | 95% CI | *P* | B | 95% CI | *P* |
| PAM | **-.27** | (-.38; -.15) | **<.001** | **-.23** | (-.36; -.11) | **<.001** |
| ComQ | **-.11** | (-.20; -.02) | **.022** | -.05 | (-.13; .04) | .314 |

***B. Sensitivity Analyses***

| Variable | 12-Month Physical Fatigue | | | 24-Month Physical Fatigue | | |
| --- | --- | --- | --- | --- | --- | --- |
|  | B | 95% CI | *P* | B | 95% CI | *P* |
| PAM | **-.14** | (-.27; -.01) | **.043** | -.10 | (-.21; .09) | .149 |
| ComQ | -.09 | (-.19; .00) | .059 | -.01 | (-.09; .11) | .835 |
| Previous physical CRF | **.40** | (.33; .47) | **<.001** | **.43** | (.36; .50) | **<.001** |
|  | 12-Month Total Fatigue | | | 24-Month Total Fatigue | | |
|  | B | 95% CI | *p* | B | 95% CI | *p* |
| PAM | **-.13** | (-.24; -.03) | **.015** | -.10 | (-.22; .01) | .078 |
| ComQ | -.06 | (-.14; .02) | .169 | .00 | (-.08; .08) | .976 |
| Previous total CRF | **.44** | (.37; .51) | **<.001** | **.43** | (.36; .49) | **<.001** |

*Note.
All results are pooled estimates of 20 multiply imputed datasets ( n = 633 at 12 months; n = 606 at 24 months). If a participant was deceased, missing values after death were not imputed. All models are adjusted for age at diagnosis, sex (female/male), education (low/high), living situation (with others/alone), number of comorbidities 4-6 months after diagnosis, cancer stage (I-II, III, IV, X), metastases 4-6 months after diagnosis (yes/no), and current status of chemo-, radio-, hormone-, and targeted/immunotherapy at the respective 12 or 24 months follow-up (never, current or recently completed, completed >6 months ago, unknown). The sensitivity analyses further adjust for previous fatigue 4-6 months after diagnosis. Effect estimates are unstandardized regression coefficients. PAM, ComQ and previous CRF are all 0-100 scales.*
